# Use of Essential Oils and Volatile Compounds as Biological Control Agents

**DOI:** 10.3390/foods10051062

**Published:** 2021-05-12

**Authors:** Caroline De Clerck, Manon Genva, M. Haissam Jijakli, Marie-Laure Fauconnier

**Affiliations:** 1 AgricultureIsLife, Gembloux Agro-Bio Tech, Liege University, Passage des Déportés 2, 5030 Gembloux, Belgium; Caroline.declerck@uliege.be; 2 Laboratory of Chemistry of Natural Molecules, Gembloux Agro-Bio Tech, Liege University, Passage des Déportés 2, 5030 Gembloux, Belgium; m.genva@uliege.be; 3 Integrated and Urban Plant Pathology Laboratory, Gembloux Agro-Bio Tech, Liege University, Passage des Déportés 2, 5030 Gembloux, Belgium; MH.Jijakli@uliege.be

Plants containing essential oils have been used for centuries as spices, remedies or for their pleasant odor. In the Middle Ages, the development of distillation techniques made it possible to obtain essential oils, which have continued to be used in their historical applications in food, medicine or cosmetics [1]. However, over the last few decades, the essential oil sector has entered a new dimension, as its fields of application are constantly increasing, largely due to the biocidal properties of its constituents.

The emergence of the resistance of targeted populations, ecological concern and impact on human health paved the way to the development of more sustainable alternatives to synthetic conventional biocides. Essential oils that combine highly biocidal properties with a specific or broad spectrum of action as well as a high volatility, thus limiting residues in foodstuff or the environment, are perfect candidates for a new generation of biocides. Used in plant protection as bactericides, fungicides or insecticides in both pre- and post-harvest treatments; as food ingredients to increase shelf-life; or incorporated in innovative packaging, research in the field of essential oils has a bright future ahead of it.

Three major subjects were discussed in the present Special Issue entitled “Use of Essential Oils and Volatile Compounds as Biological Control Agents”: stored product insecticides, plant protection and food additives-food packaging.

Six research articles were published on the first topic, focusing on the insecticidal properties of essential oils, with the challenging perspective of replacing chemical insecticides that are widely used during crop cultivation and post-harvest treatments and therefore reducing the quantities of residues in foods. Oftadeh et al. first highlighted the high level of interest in essential oil from flowers of *Artemisia annua* L. in the control of *Glyphodes pyloalis* Walker, which damages mulberry leaves and induces the transmission of plant pathogenic agents [2]. In the second paper, the authors described the interesting contact toxicity of *Satureja intermedia* C.A.Mey essential oil against *Aphis nerii* Boyer de Fonscolombe, which is an insect pest in many ornamental plant cultures causing direct plant damage and transmitting pathogenic viruses. Interestingly, *Coccinella septempunctata* L., which is a predator of *A. nerii* and is used as biocontrol agent, was less susceptible to the essential oil. Moreover, the authors also described the elevated fumigant toxicity of *S. intermedia* essential oil against *Trogoderma granarium* Everts, *Rhyzopertha dominica* Fabricius, *Tribolium castaneum* Herbst, and *Oryzaephilus surinamensis* L., which are all common insect pests in stored products [3]. Loss during food storage due to insect infestation is a huge problem, both in developing and in developed countries. Contact chemical insecticides are therefore traditionally used to reduce food losses, with the problems of resistance appearance and the persistence of chemical residues in food. Essential oils, along with their complex composition, their low mammal toxicity and their high volatility, have emerged as promising alternatives to chemical insecticides in stored products. In the next article, Demeter et al. studied the insecticidal activity of 25 essential oils against *Sitophilus granarius* L., which is one of the main insect pests during grain storage. The authors showed a high potential in different essential oils, such as those from *Allium sativum* L., *Mentha arvensis* L. and *Eucalyptus dives* Schauer for the control of *S. granarius* in stored products [4]. Tanoh et al. also showed the toxicity of the newly described essential oils from *Zanthoxylum leprieurii* Guill. & Perr. against the same insect [5]. Thereafter, Owolabi et al. described the high toxicity of essential oils from a Nigerian plant, *Launaea taraxacifolia* (Willd.) Amin ex C. Jeffrey, against *Sitophilus oryzae* L., the rice weevil that causes high food losses during grain storage [6]. Finally, Liang et al. showed the high insecticidal properties of essential oil from *Elsholtzia ciliata* (Thunb.) Hyl. and of its major components, carvone and limonene, in the control of *Tribolium castaneum* Herbst, a common beetle affecting many stored products, such as cereals and flours [7].

In the second topic of the present Special Issue, two articles described the high interest of essential oils in crop plant protection. De Clerck et al. firstly screened 90 commercially available essential oils for their in vitro antifungal and antibacterial activities against 10 phytopathogens that particularly attack plant crops and decrease food production yields. The authors highlighted that several essential oils, such as that from *Allium sativum* L., are active on diverse pathogens and thus have a “generalist” effect, while other essential oils such as that from *Citrus sinensis* (L.) Osbeck have an action on one to three pathogens, and thus a more “specific” effect [8]. In the review from Werrie et al., the authors described the high interest of essential oils for the development of biopesticides, but they also underlined the different restrictions on their use, as some of them display phytotoxicity on untargeted crops. The authors mentioned the different parameters that need to be taken into account to limit that risk, such as the mode of application, the phenological state and the product formulation [9].

In the last topic of this Special Issue, different authors studied the potential of essential oils as food additives or for their incorporation into food packaging. Siroli et al. firstly showed that the incorporation of essential oils into the marinade increased the sensorial perception of the marinated pork loin [10]. In the next article, Licon et al. showed that the incorporation of essential oils from *Thymus vulgaris* L. in milk used for the production of pressed ewes’ cheese had an interesting antimicrobial effect, with a decrease in the growth of exogenous detrimental microorganisms without affecting the cheese natural flora [11]. Ben-Fadhel et al. then highlighted the antimicrobial interest of essential oils for the treatment of ready-to-eat carrots. Indeed, their incorporation into emulsions that were applied to the carrot surface allowed the lengthening of the carrot shelf-life by two days [12]. Ruengvisesh et al. studied the antimicrobial activities of micelles formed from sodium dodecyl sulfate. The authors showed that eugenol-loaded micelles were particularly effective in inhibiting *Escherichia coli* and *Salmonella enterica* when applied on fresh spinach surfaces [13]. Essential oils also emerged as interesting bioactive additives for their incorporation into active packaging. In their article, Díaz-Galindo et al. showed that the incorporation of cinnamon essential oil emulsions into thermoplastic starch leads to a decrease in the growth rate of *Botrytis cinerea* without affecting the thermal stability of the packaging [14]. As essential oil volatility may limit their applications when the release is too fast, Maes et al. studied the potential of biosourced dendrimers to encapsulate essential oils. Their results show that stirring time and stirring rate are crucial parameters that need to be optimized for an efficient encapsulation, which paves the way to numerous essential oil applications when a slower release is needed [15]. Bleoancă et al. studied two different treatments for the formation of edible films containing thyme extracts. Both high-pressure-thermally treated films and thermally treated ones display different structures with different abilities to retain volatile compounds [16]. Finally, Kostoglou et al. showed the promising potential of three plant terpenoids—carvacrol, thymol and eugenol—as anti-biofilms agents, as they showed significant anti-biofilm activities against *Staphylococcus aureus* and *Staphylococcus epidermidis*. Those two microorganisms are notably the cause of foodborne diseases and nosocomial infections [17].

The success of this Special Issue demonstrates clearly the scientific interest around the use of volatile compounds, especially essential oils, as biological control agents in food products. In addition, with controversial products being removed from the market, alternative products such as essential oils are expected to rise.

While this topic seems to have a bright future, some questions and difficulties remain. One of the first challenges encountered in the development of biopesticides using volatile molecules is their short persistence (volatility, degradation, etc.) in comparison to synthetics. This can be positive in terms of environmental impacts and in terms of food residues, but the release kinetic of the compounds and their molecular dynamics have to be known and controlled to ensure the product’s efficacy. The formulation thus plays an important role, and technology is evolving, as highlighted in several papers of this Special Issue, with the development of nano-emulsions and encapsulation, among others. These formulations are also important to avoid the apparition of any adverse tastes or odors on stored food products. The authors also pointed out the need for an upscaling of the tests, which will help to assess the practical applicability of the treatments. A number of compounds have proven their efficacy in vitro and seem promising. However, in vitro tests will always need to be confirmed in vivo.

Essential oils and volatile compound activities are often attributed to mixtures of compounds. While this could be an advantage to prevent the development of resistances if they present different modes of action, as has been shown in [18], with two constituents of essential oils with distinct chemical structure interacting differentially with plant plasma membrane, this complex composition presents challenges to regulatory standards, where regulations are generally designed for synthetic substances that contain a single, highly concentrated and persistent molecule. This is leading to difficulties regarding market approval by the different regulatory agencies throughout the world, as well as economic considerations. Even if procedures are sometimes available for plant-based products, few active substances have been registered so far, especially in the pre- and post-harvest fields. Uses as ingredients in food products are less problematic, as only a few essential oils have restricted regulation concerns (e.g., mint essential oils).

More investigations need to be performed to decipher the mechanism of action of these volatile compounds, including the role of minor components and the synergic or additive effect among them. This will be crucial to evaluate the risks on the environment (plants, beneficial organisms (insects, worms …), soil microbiota, etc.), and human health, as well as to secure their industrial use.

To conclude, the use of volatile compounds and essential oils in particular for sustainable agricultural practices or as food ingredients seems promising, and extensive research will probably clarify or deny their relevance in diverse applications. They can be an efficient alternative to synthetic plant protection products when properly formulated and integrated with other pest management strategies; they can also be valuable food ingredients or innovative packaging constituents

The works collected in this Special Issue will certainly contribute to the field by increasing the knowledge on volatile compounds used as biological control agents, their efficiency and formulation in a large panel of situations related to the food sector.
